# 2123. The Impact of *Staphylococcus aureus* Peri-transplant Cultures on Six-Month Outcomes in Lung Transplantation

**DOI:** 10.1093/ofid/ofac492.1744

**Published:** 2022-12-15

**Authors:** Courtney E Harris, Abhinav Mehta, Andy J Kim, Hilary J Goldberg, Hari R Mallidi, Hari R Mallidi, Keri Townsend, Antonio Coppolino, Tany Thaniyavarn, John C Kennedy, Stefi F Lee, Valerie Durney, Shirley Marshall, Jessica Fenty-Scotland, Lisa A Cosimi, Victor Kovac, Nicolas C Issa, Nirmal S Sharma, Lindsey R Baden, Ann E Woolley

**Affiliations:** Brigham & Women's Hospital, Boston, Massachusetts; Brigham & Women's Hospital, Boston, Massachusetts; Brigham and Women's Hospital, Boston, Massachusetts; Brigham and Women's Hospital, Boston, Massachusetts; Brigham and Women's Hospital, Boston, Massachusetts; Brigham and Women's Hospital, Boston, Massachusetts; Brigham & Women's Hospital, Boston, Massachusetts; Brigham and Women's Hospital, Boston, Massachusetts; Brigham and Women's Hospital, Boston, Massachusetts; Brigham and Women's Hospital, Boston, Massachusetts; Brigham and Women's Hospital, Boston, Massachusetts; Brigham & Women's Hospital, Boston, Massachusetts; Brigham & Women's Hospital, Boston, Massachusetts; Brigham & Women's Hospital, Boston, Massachusetts; Brigham & Women's Hospital, Boston, Massachusetts; Brigham & Women's Hospital, Boston, Massachusetts; Brigham & Women's Hospital, Boston, Massachusetts; Brigham and Women's Hospital, Boston, Massachusetts; Brigham and Women's Hospital, Boston, Massachusetts; Brigham and Women's Hospital, Boston, Massachusetts

## Abstract

**Background:**

*Staphylococcus aureus* (*S. aureus*) infections post-lung transplant lead to increased mortality. The impact of *S. aureus* peri-transplant respiratory cultures on post-transplant outcomes is unknown as is the optimal duration of peri-transplant antibiotics. We compared lung transplant recipients with and without *S. aureus* growth on peri-transplant cultures and the impact on 6-month outcomes including rejection, survival, and occurrence of *S. aureus* infections.

**Methods:**

A single-center, retrospective study of lung transplant recipients between January 2017 and April 2021 was performed. Donor/recipient characteristics, microbiologic data, antibiotics, and 6-month outcomes were analyzed.

**Results:**

In this 4.5-year study period, 229 patients underwent lung transplant. 121 (53%) had *S. aureus* growth on peri-transplant cultures, of which 84 (69%) were methicillin-susceptible (MSSA), 30 (25%) methicillin-resistant (MRSA), and 7 (6%) had MSSA and MRSA. Median duration of antibiotics post-transplant was 28-days in the *S. aureus* group. 67% of non-*S. aureus* recipients received *S. aureus* targeting antibiotics (p = < 0.001) for other indications for a median of 13-days. In the *S. aureus* cohort, donors were younger (median age 33 vs 38 years, p = 0.027) and more donors died from drug intoxication (50% vs 31%, p = 0.005) [**Table 1**]. Recipient baseline characteristics and median length of hospitalization were similar in both cohorts. There were no statistically significant differences in 6-month outcomes, including rates of rejection or survival [**Figure 1**]. Having grown *S. aureus* in peri-transplant cultures was not associated with a higher incidence of post-transplant *S. aureus* infections (10% vs 13%, p = 0.534). Patients who had a *S. aureus* infection post-transplant had lower 6-month survival that was not statistically significant (85% vs 93%, p = 0.131).

**Figure:**
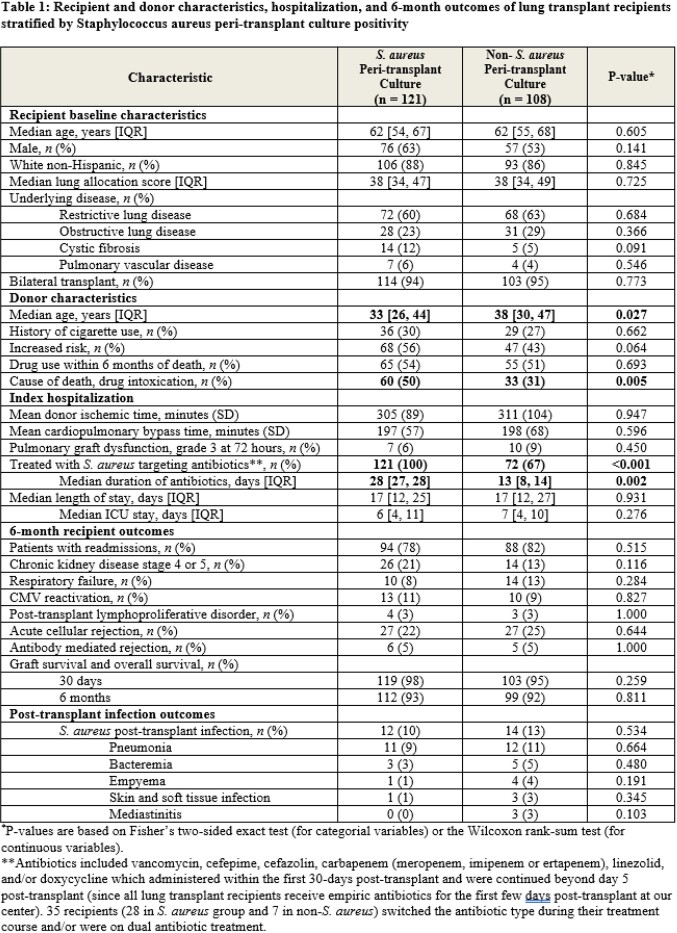

**Figure:**
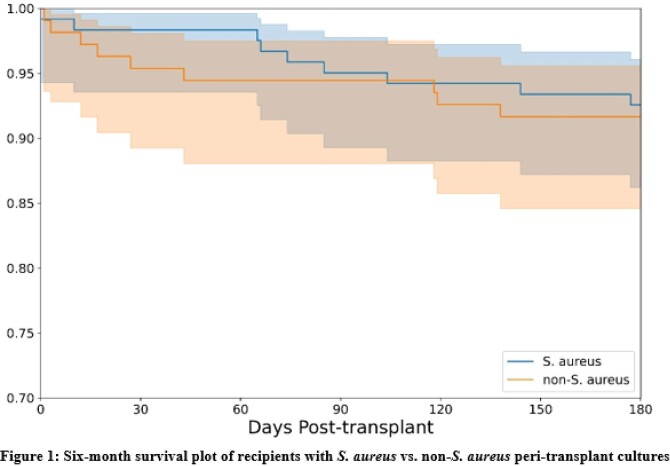

**Conclusion:**

The growth of *S. aureus* on peri-transplant respiratory cultures with a median 4-week antibiotic course does not increase the risk of having a *S. aureus* infection post-transplant and is not associated with increased mortality or rejection at 6-months. The impact of a shorter duration of peri-transplant antibiotics on recurrence of infection and outcomes needs to be further studied.

**Disclosures:**

**Nicolas C. Issa, MD**, AiCuris: Grant/Research Support|Merck: Grant/Research Support.

